# Can Protected Areas Mitigate Lyme Disease Risk in Fennoscandia?

**DOI:** 10.1007/s10393-019-01408-4

**Published:** 2019-04-08

**Authors:** Julien Terraube

**Affiliations:** 1 Global Change and Conservation Lab, Organismal and Evolutionary Biology Research Program, Faculty of Biological and Environmental Sciences, University of Helsinki, PO Box 65, Viikinkaari 1, 00014 Helsinki, Finland; 2 HELSUS, Faculty of Biological and Environmental Sciences, University of Helsinki, Helsinki, Finland

**Keywords:** Trophic cascades, Tick-borne disease, Global change, Top predator, Conservation, Boreal ecosystems

## Abstract

This Forum article synthesizes the current evidence on the links between predator-prey interactions, protected areas and spatial variations in Lyme disease risk in Fennoscandia. I suggest key research directions to better understand the role of protected areas in promoting the persistence of diverse predator guilds. Conserving predators could help reducing host populations and Lyme disease risk in northern Europe. There is an urgent need to find possible win-win solutions for biodiversity conservation and human health in ecosystems facing rapid global environmental change.

## Introduction

Lyme disease (LD) is the most common tick-borne disease in temperate forested regions of North America and Eurasia, with increased number of reported cases worldwide (Stone et al. 2017). LD is caused by some members of the *Borrelia burgdorferi* sensu lato (s.l.) species complex, carried and transmitted by several species of *Ixodes* ticks, the most common in Europe being *Ixodes ricinus* (de la Fuente et al. 2008). All *Ixodes* tick species have three feeding stages (larvae, nymph and adult), which take a single blood meal from a wide range of hosts before molting to the next stage (larvae and nymphs, the latter being responsible for the majority of human cases of LD), or reproducing and dying (adult females) (Kilpatrick et al. 2017a). In Europe, host species include > 40 vertebrate species, three main taxonomic groups (i.e., small rodents, passerine birds and ungulates) being responsible for maintaining populations of *I. ricinus* and *Borrelia burgdorferi* s.l. (Hofmeester et al. 2016).

LD is expanding fast as a result of climate and land-use change, both causing tick and host expansion (Lindgren et al. 2000; Medlock et al. 2013). However, the impact of changing trophic interactions on disease dynamics through cascading effects on host abundance has received less attention in Europe than in North America (Levi et al. 2012, 2016), and little is known regarding the effect of land protection status on the relationship between ecological networks and disease risk (Terraube et al. 2017). Protected areas (PAs) may influence the abundance of hosts and their predators which could impact local *Ixodes* tick abundance and LD risk (defined here as the density of questing infected nymphs in the environment, see Kilpatrick et al. 2017a).

Northern Europe offers a unique setting to explore the interactions among LD, trophic interactions, PAs and global change because: (1) Increasing incidence of LD cases have been reported in Finland (Sajanti et al. 2017), Norway (Mysterud et al. 2016) and Sweden (Bennet et al. 2006); (2) rapid climate change in high-latitude regions (Bärring et al. 2017) could facilitate the spread of LD further north; (3) intensification of forestry management and agricultural practices have extensively altered boreal landscapes in recent decades (Bradshaw et al. 2009); (4) host populations have increased as a result of these global environmental changes (Jaenson et al. 2018); (4) particularly diverse top- and mesopredator (TP and MP, respectively) guilds can exert top-down control on host communities (Ostfeld et al. 2018); and (5) a combination of anthropogenic pressures and ecological factors (Sundell et al. 2004) drives spatial variation in the composition of predator guilds, constituting landscape-scale natural experiments.

In the following sections, I will highlight (1) host population trends in the study area; (2) how trophic interactions could affect these main host species; finally (3) how PAs may impact predator abundance, host species and thus tick abundance and LD risk.

## Host Population Trends in Fennoscandia

Insectivores (particularly *Sorex araneus*) and small rodents (*Microtus* and *Myodes* voles and *Apodemus* mice) are the most important hosts of larval *I. ricinus* in Fennoscandia (Mysterud et al. 2016; Tälleklint and Jaenson 1994). Population cycles of small rodents have recently dampened in northern Europe (Cornulier et al. 2013). The reasons for these large-scale changes remain poorly understood but, in Lapland, climate change plays a role in limiting vole population growth rates (Terraube et al. 2015). However, shifts in rodent community composition (e.g., increased contribution of an important reservoir host, the bank vole (*Myodes glareolus*), see Ecke et al. 2017) may be more important than overall abundance in explaining variation in LD incidence (LoGiudice et al. 2003).

Wild ungulates (e.g., *Capreolus capreolus* and *Alces alces*) and lagomorphs (*Lepus timidus* and *L. Europaeus*) are important feeding hosts for adult *Ixodes* ticks (Jaenson et al. 2009; Mysterud et al. 2016; Tälleklint and Jaenson 1994). Overall, cervid populations are increasing in northern Europe, as a result of milder winters and adaptation to forest fragmentation (Burbaite and Csányi 2009; Kekkonen et al. 2016). In southern Sweden, higher deer densities have been shown to result in higher tick abundance and increase the incidence of tick-borne diseases (Jaenson et al. 2018).

Finally, passerine birds (but also lagomorphs) are known to be key hosts for nymphal ticks in Europe (Taragel’ová et al. 2008). However, scant information is currently available on the role of different bird species in the natural cycle of *B. burgdorferi* s.l. in Fennoscandia.

The role of top- and mesocarnivores as secondary tick hosts is probably negligible in Europe (Hofmeester et al. 2018).

## Functional Role of Predators and Potential Consequences for the Main LD Host Species

**Fig. 1:**
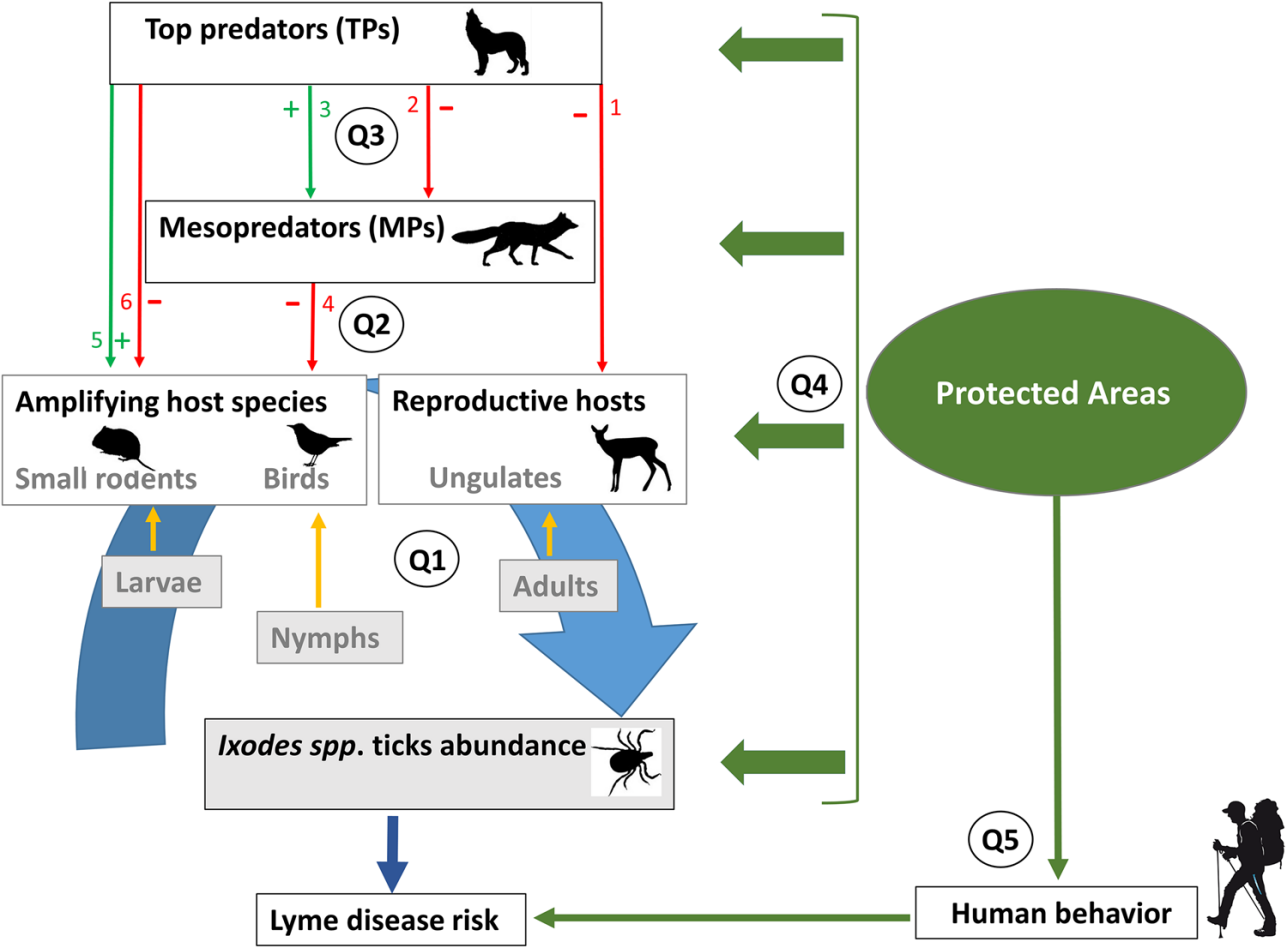
Diagram of hypothesized three-/two-level trophic cascades in European boreal ecosystems linking top predator (TP) to mesopredator (MP) and host (amplifying and reproductive) communities. Host abundance finally influences *Ixodes* tick abundance, at the three different stages, each stage feeding primarily on a specific host group (adult female ticks need a blood meal on a large vertebrate to reproduce, mostly ungulates, while larvae and nymphs target preferentially rodents and passerine birds, respectively; Hofmeester et al. 2016), which result in spatial variation in Lyme disease risk. Red arrows associated with negative signs describe a negative effect of higher trophic level species on the predator or prey populations of lower trophic levels. Green arrows associated with positive signs describe a positive effect of higher trophic level species on the predator or prey populations of lower trophic levels. Arrow 1 illustrates the top-down control of top predators on ungulate populations that may vary depending on local context. Arrow 2 describes the potential control that top predators exert on mesopredator populations. Arrow 3 illustrates facilitative effects that top predators may have on mesopredators through resource provisioning. Arrow 4 represents the top-down control that mesopredators exert on host species. Arrows 5 and 6 show either positive or negative indirect effects that top predators may have on host species depending on the outcome of interspecific interactions between top- and mesopredators (see arrows 2 and 3). Q1, Q2, Q3, Q4 and Q5 highlight important research question that are developed in the text (see Conclusion section). These research questions need to be addressed at each trophic level to better understand the mechanistic linkages connecting the structure and composition of predator communities to Lyme disease risk in humans

Predator communities can impact LD risk directly and indirectly by: (1) reducing the most important host species abundance or changing the host community composition and (2) inducing fear-mediated changes in habitat use of the main host species, which could decrease LD transmission risks (Hofmeester et al. 2017; Keesing et al. 2006; Ostfeld and Holt 2004). Avian and mammalian TPs can influence the abundance of small mammals either positively or negatively, depending on their effects on mesopredator populations (Fig. 1). These TPs could release the predation pressure on small mammals by negatively influencing MP populations (Levi and Wilmers 2012; Ritchie and Johnson 2009), potentially increasing LD risk. Alternatively, large carnivores can control ungulate populations (Andrén and Liberg 2015), potentially decreasing the abundance of reproductive hosts for adult female ticks. To date, the balance of these complex trophic pathways (three-versus two-level trophic cascade) in terms of local LD risk (Fig. 1) remains poorly understood in Europe. Finland, Norway and Sweden are the only countries in Europe where four species of large carnivores (Brown bear *Ursus arctos*, Eurasian lynx *Lynx lynx*, Gray wolf *Canis lupus* and Wolverine *Gulo gulo*) are still present, offering a unique opportunity to study intra-guild relationships between these species, their potential impact on mesocarnivore abundance (suppression/facilitation patterns depending on local context; Elmhagen and Rushton 2007; Khalil et al. 2014) and their cascading effects on host prey species, tick abundance and infection prevalence in ticks and hosts.

As in other parts of Europe, large carnivore populations have partly recovered lost ground in northern Europe during the last decades (Chapron et al. 2014). However, more recently, population recovery of some of these large carnivores has been slowing down. For example, the recovery of gray wolf in Fennoscandia has been reduced due to intense human–carnivore conflict (Liberg et al. 2012; Suutarinen and Kojola 2017). Aside from direct persecution, anthropogenic disturbance has also been shown to limit the functional role of large carnivores in boreal ecosystems (van Beeck Calkoen et al. 2017). In Fennoscandia, the red fox (*Vulpes vulpes*) is a common prey of the lynx (Elmhagen et al. 2010). There is increasing evidence that both wolf and lynx can limit red fox populations (Pasanen-Mortensen et al. 2013; Wikenros et al. 2017). However, these TPs could also facilitate MP populations through carcass provisioning (Sivy et al. 2017).

Previous research has shown that, in Fennoscandia, specialist and generalist MPs impact small mammals’ abundance in different ways, the former causing strong fluctuations in rodent abundance (Hanski et al. 2001), and the latter along with nomadic avian predators regulating prey abundance and potentially disease incidence at consistently lower levels (Erlinge et al. 1983; Khalil et al. 2016a, b; Lindström et al. 1994). Therefore, further research should investigate the respective role of specialist and generalist predators in limiting small mammal densities and movements, along gradients of varying anthropogenic pressure and how this impacts LD risk.

## Do PAs Maintain Diverse Predator Communities in Fennoscandia with Cascading Effects on Infection Prevalence and LD Risk for Humans?

PAs are the backbone of biodiversity conservation worldwide (Gray et al. 2016). However, the success of this conservation approach can be compromised by external threats and poor management, calling for systematic quantification of the effectiveness of PAs (Watson et al. 2016). In a context of increased zoonotic disease transmissions in degraded ecosystems, further research is needed to understand if biodiversity conservation interventions (e.g., the restoration of PAs) may be a potential win-win strategy for maintaining ecosystem health and protecting public health (Bauch et al. 2015; Kilpatrick et al. 2017b; Terraube et al. 2017).

Indeed, PAs could help mitigate LD risk in Fennoscandia by maintaining forest complexity and continuity (Allan et al. 2003) and preserving healthier and more diverse predator communities, both factors decreasing host abundance and the probability of contact between ticks and their hosts (‘habitat dilution’, see Ehrmann et al. 2018).

However, while few studies have examined the effectiveness of this fragmented PAs network in conserving forest patches harboring high biodiversity (Virkkala and Rajasärkka 2007), the effectiveness of PAs in maintaining large carnivore populations remains highly debated (Rauset et al. 2016). In order to understand the influence of PAs on LD risks for humans, it is hence critical to study how protection status influences the main host species and their predators (Fig. 1, Millins et al. 2017). Such research can have direct implication for environmental policies and PA management (e.g., carnivore hunting regulation inside PAs).

Considering the frequency of human visits to forests is essential to predict the spatial distribution of tick-borne diseases (Rizzoli et al. 2011; Vanwambeke et al. 2010). Outdoor activities are extremely popular in northern Europe, especially in Finland, where PAs have been increasingly visited (Puhakka and Saarinen 2013). When evaluating the overall contribution of these PAs to LD risk for humans, special consideration should hence be given to the growing human presence during the questing period (June–July), which may boost the risk of contact with infected ticks.

### Research Agenda to Investigate the Links Between Protected Areas, Composition of Host and Predator Communities and LD risk

Developing a comprehensive understanding of the community ecology of LD in Fennoscandia and of the potential effects of PAs on its incidence requires addressing the following questions:

- Host species for *Ixodes* ticks:
  What is the relative importance of the different groups of host species for *Ixodes* ticks across their life cycle (Q1 in Fig. 1)?
  How do spatiotemporal variations in co-occurrence and abundance of different hosts influence overall tick abundance and LD risk (Q1 in Fig. 1)?
  How do land-use gradients and land protection status influence spatial variation in the importance of these different host groups for *Ixodes* ticks?
- Multi-level trophic cascades and LD risk:
  Do TPs mainly facilitate or suppress MPs (Q3 in Fig. 1)?
  Do MPs and TPs affect host species and the density of infected nymphs through direct or indirect pathways and what is the overall effect of TPs on LD risk? (Q2 in Fig. 1)?
  How does land protection status and other environmental drivers (climate, habitat composition) influence the trophic pathways identified above and impact LD risk at various scales (Q4 in Fig. 1)?
- Human behavior, protected areas and LD incidence:
  How does human behavior and connectedness to nature interact with the multi-level trophic cascades identified above to influence LD incidence at various scales?
  Do protected areas buffer or amplify LD risk and incidence considering both interactive effects between changes in wildlife communities and human behavior (Q5 in Fig. 1)?

Successful implementation of this research agenda requires establishing multi-disciplinary teams (wildlife ecologists, epidemiologists, public health experts, environmental practitioners and behavioral research scientists) and combining empirical, experimental and modeling approaches at local and global scales. Existing nation-wide datasets of LD incidence and wildlife community composition can be used in several northern European countries to assess the environmental drivers of LD incidence. Comparative designs inside and outside PAs, where host abundance, predator community structure, tick density and infection prevalence in ticks and hosts are sampled, would help determine how multi-level trophic cascades influence LD risk in boreal ecosystems.

In conclusion, it is time for research to strive to understand the top-down processes regulating LD transmission in Fennoscandia. Such research could improve public attitude toward predators and provide powerful motivation for society to preserve complex ecological networks in boreal ecosystems currently facing the combined effects of land cover change and climate change.
